# Effects of cipepofol on breathing patterns, respiratory drive, and inspiratory effort in mechanically ventilated patients

**DOI:** 10.3389/fmed.2025.1539238

**Published:** 2025-02-25

**Authors:** Rui Su, Linlin Zhang, Yu-Mei Wang, Ming-Yue Miao, Shuya Wang, Yong Cao, Jian-Xin Zhou

**Affiliations:** ^1^Department of Critical Care Medicine, Beijing Tiantan Hospital, Capital Medical University, Beijing, China; ^2^Department of Critical Care Medicine, Beijing Shijitan Hospital, Capital Medical University, Beijing, China; ^3^Haisco Pharmaceutical Group Co. Ltd., Chengdu, China; ^4^Clinical and Research Center on Acute Lung Injury, Emergency and Critical Care Center, Beijing Shijitan Hospital, Capital Medical University, Beijing, China

**Keywords:** cipepofol, sedation, breathing pattern, respiratory drive, inspiratory effort

## Abstract

**Background:**

Cipepofol is a highly selective gamma-aminobutyric acid A receptor potentiator. As a new sedative drug, detailed studies on its respiratory effects are further needed. The present study aims to investigate the effects of cipepofol on breathing patterns, respiratory drive, and inspiratory effort in mechanically ventilated patients.

**Methods:**

In this one-arm physiological study, cipepofol was initiated at 0.3 mg/kg/h and increased by 0.1 mg/kg/h every 30 min until reaching 0.8 mg/kg/h. Discontinuation criteria were Richmond Agitation and Sedation Scale (RASS) score ≤ −4 or respiratory rate (RR) < 8 breaths/min or pulse oxygen saturation (SpO_2_) < 90%. The primary outcomes were changes from baseline in respiratory variables [RR, tidal volume (VT), minute ventilation (V_min_), airway occlusion pressure at 100 msec (P_0.1_), pressure muscle index (PMI), expiratory occlusion pressure (P_occ_)] at 30 min after 0.3 mg/kg/h cipepofol infusion. The secondary outcomes included changes in respiratory variables, cardiorespiratory variables, and RASS scores at rates of cipepofol from 0.3 to 0.8 mg/kg/h.

**Results:**

20 patients were enrolled and all of them completed the cipepofol infusion rate at 0.3 mg/kg/h, achieving RASS score of −2 to +1. For the primary outcomes, there was a significant reduction in VT (390.9, [356.6–511.0] vs. 451.6 [393.5–565.9], *p* = 0.002), while changes in RR (16.7 ± 2.7 vs. 16.2 ± 3.4, *p* = 0.465) and V_min_ (7.2 ± 1.8 vs. 7.5 ± 1.9, *p* = 0.154) were not significant. The reductions in P_0.1_ (*p* = 0.020), PMI (*p* = 0.019), and P_occ_ (*p* = 0.007) were significant. For secondary outcomes, as the infusion rate of cipepofol increased from 0.3 to 0.8 mg/kg/h, there was a further decrease in VT (*p* = 0.002) and an increase in RR (*p* < 0.001), while the change in V_min_ (*p* = 0.430) was not significant. RASS score (*p* < 0.001) was further decreased.

**Conclusion:**

Cipepofol demonstrates the capability to achieve RASS score −2 to +1 in mechanically ventilated adult patients. The effect of cipepofol on breathing patterns was a decrease in VT, while changes in RR and V_min_ were insignificant. The effect on respiratory drive and inspiratory effort significantly reduced P_0.1_, PMI, and P_occ_.

**Clinical trial registration:**

ClinicalTrials.gov, identifier NCT06287138. https://clinicaltrials.gov/study/NCT06287138

## Introduction

Critically ill patients often experience noxious stimuli from endotracheal tubes, artificial ventilation, and other intensive care procedures such as bronchial suctioning, physiotherapy, and catheter placement (1, 2). Proper administration of analgesia and sedatives is crucial in the care of mechanically ventilated patients which relieves pain and anxiety, reduces stress, and prevents agitation-related harm (3). The paradigm of eCASH has established best practices in sedation management, emphasizing early comfort using analgesia, minimal sedatives, and maximal human care (4). However, sedative drugs come with potential adverse effects such as nausea, vomiting, and especially respiratory depression (5), which may increase the risk of complications and prolong the clinical course (6, 7). Consequently, this often leads to inappropriate use of such agents, sometimes with doses higher or lower than those required for adequate therapeutic effect (8). Hence, there is a great interest in measuring the respiratory effects of commonly used drugs, such as propofol (9, 10) and remifentanil (11), as well as assessing new agents in clinical settings.

**Figure 1:**
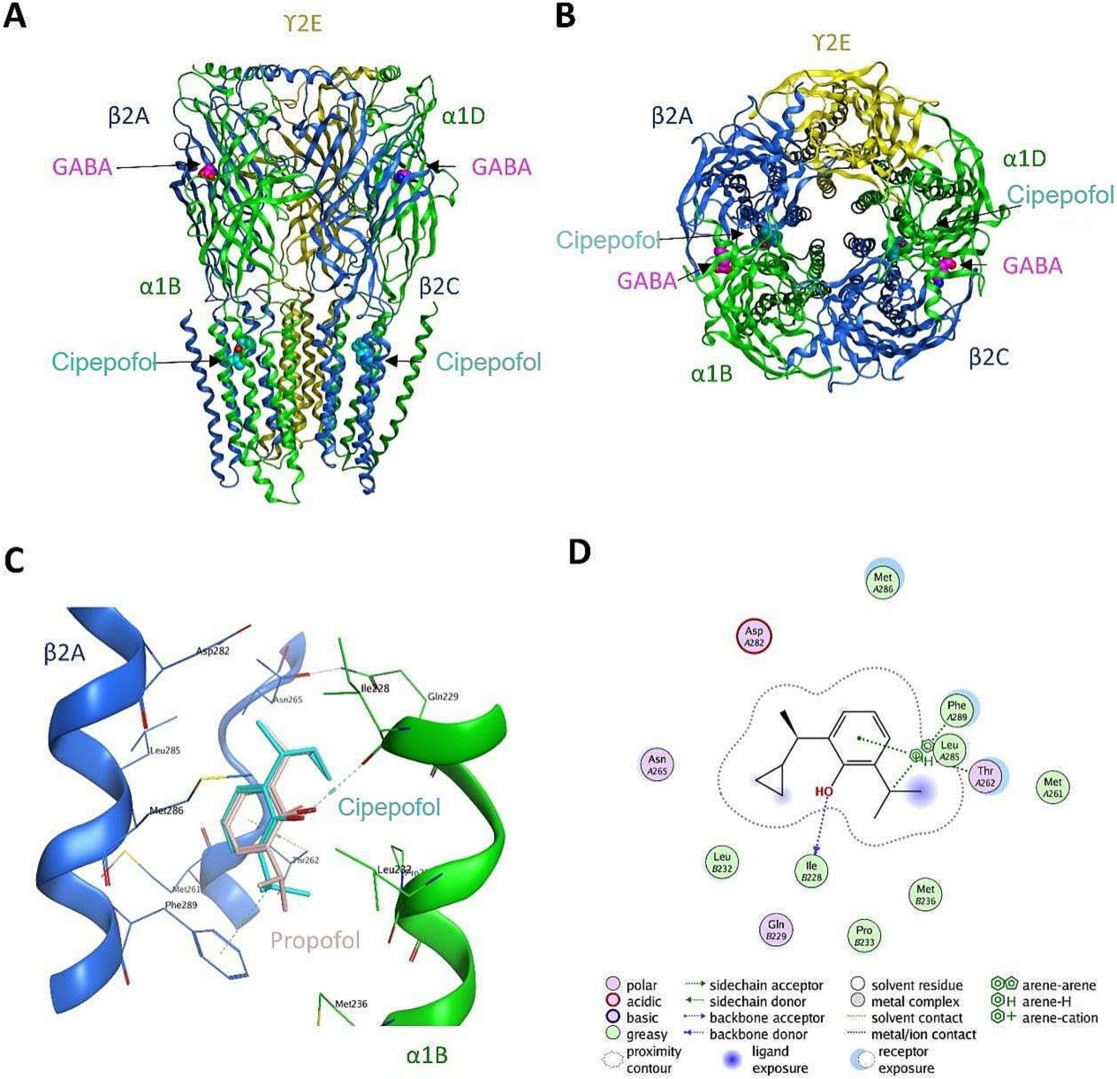
The putative interactions between GABA_A_ receptor and cipepofol. **(A)** The front-view of cipepofol binding to the cavity between β_2_ subunit (blue) and α_1_ subunit (green). **(B)** The top-view of cipepofol binding to the cavity between β_2_ subunit (blue) and α_1_ subunit (green). **(C)** Hydrophobic packing was believed to be the major interaction between GABA_A_ receptor and cipepofol, while a hydrogen bond was formed between Ile228 of α_1_ subunit and the hydroxyl group in cipepofol. The propofol molecule was also shown as reference (pink). **(D)** The 2D interaction diagram of GABA_A_-cipepofol.

Cipepofol (also known as ciprofol and HSK3486) was a structural analog of propofol. As a novel 2, 6-disubstituted phenol derivative, a cyclopropyl group was incorporated into the 2,6-side chain to increase its lipophilicity, and chiral centers were introduced to break the structure symmetry (12). Cipepofol produces the hypnotic effect mainly by enhancing gamma-aminobutyric acid type A (GABA_A_) receptor-mediated inhibitory synaptic currents (13), exhibiting about four to five times the potency of propofol (14, 15). The putative interactions between GABA_A_ receptor and cipepofol are illustrated in Figure 1. Otherwise, like propofol, cipepofol produces rapid-onset action and clear wake-up with similar pharmacokinetic characteristics of absorption, distribution, and metabolism (13).

In the phase II and III clinical trials, the tolerability and sedation characteristics were comparable between cipepofol and propofol in mechanically ventilated patients (16, 17). Cipepofol induced a milder reduction in mean arterial pressure (MAP) than propofol (18), and it poses a lower risk of respiratory depression (19). However, despite these findings, as a new sedative drug, detailed studies on its adverse effects, particularly its impact on respiration are still warranted. Until now, the effects of cipepofol on breathing patterns and respiratory drive during intensive care unit (ICU) sedation have not been well-described. Therefore, the present study aimed to investigate the effects of cipepofol on breathing patterns, respiratory drive, and inspiratory effort in mechanically ventilated patients.

## Materials and methods

### Study design

The study was a single-center, prospective, physiological trial. This study was approved by the Institutional Review Board of Beijing Tiantan Hospital, Capital Medical University (KY2023 − 182-03). According to the Declaration of Helsinki, written informed consent was obtained from all patients or their legal representatives. The study was registered at ClinicalTrials.gov (NCT06287138), https://clinicaltrials.gov/study/NCT06287138.

### Patients

Patients were consecutively recruited from December 2023 to February 2024. The inclusion criteria were critically ill adults with endotracheal intubation who received mechanical ventilation in pressure support mode after surgery under general anesthesia, and the patients were expected to receive sedation for a target of Richmond Agitation and Sedation Scale (RASS) score of −2 to +1. The exclusion criteria included age less than 18 years; body mass index (BMI) less than 18 or greater than 30 kg/m^2^; pregnancy or lactation; brain stem tumors, myasthenia gravis, or neuromuscular diseases; acute severe neurological disorder or any other condition interfering with RASS assessment; systolic blood pressure (SBP) less than 90 mmHg after appropriate fluid resuscitation; heart rate (HR) less than 50 beats per minute or second- or third-degree atrioventricular block without a pacemaker; contraindications or allergies to any study medications; acute hepatitis or serious hepatic dysfunction (Child-Pugh class C); chronic kidney disease with glomerular filtration rate less than 60 mL/min/1.73m^2^.

### Study protocol

**Figure 2:**
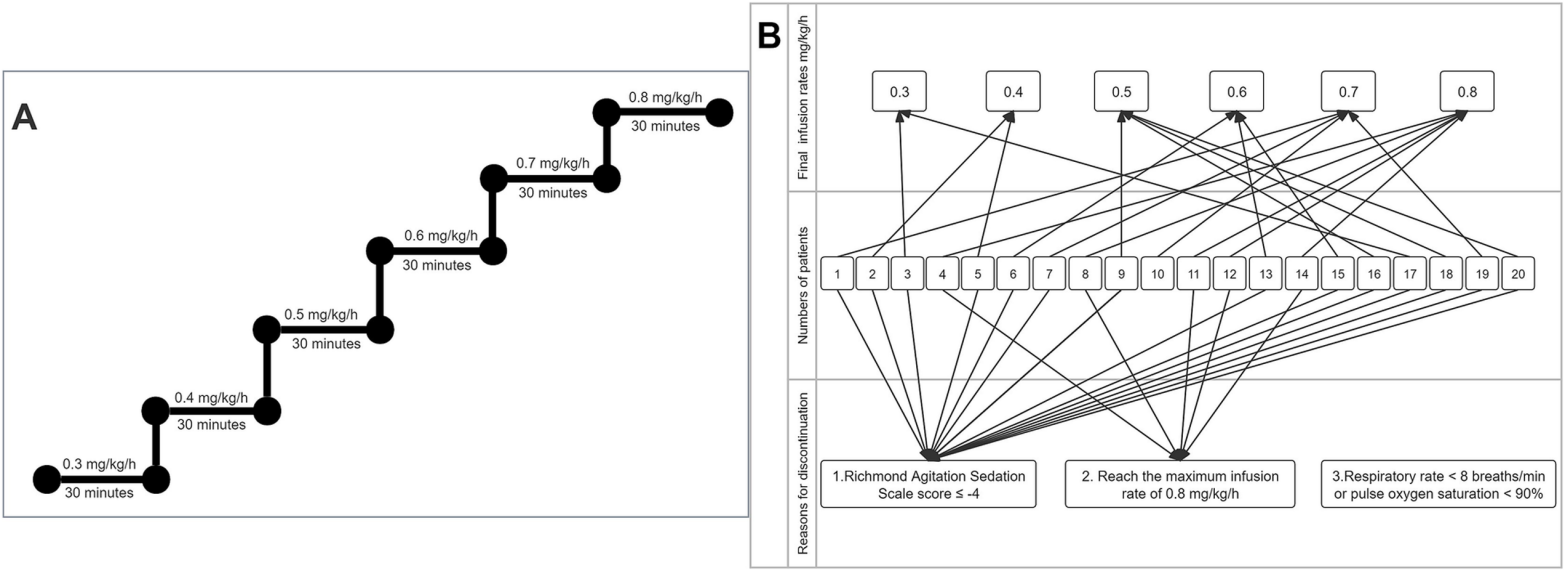
Process of cipepofol infusion. **(A)** Titration method of cipepofol. **(B)** Reasons for discontinuation and final rates are given.

During the intervention period, when the patient’s baseline sedation level had reached a RASS score ≥ −2, cipepofol was given and initiated at 0.3 mg/kg/h, which dose was increased by 0.1 mg/kg/h every 30 min, until the maximal dose of 0.8 mg/kg/h, the titration method for cipepofol is described in Figure 2A. The predefined discontinuation criterion of the study was the maximal dose of cipepofol at 0.8 mg/kg/h, RASS score ≤ −4, respiratory rate < 8 breaths/min (20), or pulse oxygen saturation (SpO_2_) < 90% (21), whichever comes the first. RASS score (22, 23) was assessed before starting the infusion and 30 min after each increase in cipepofol rate, or more often if a fluctuation in the level of sedation was observed. The reason for discontinuation and the final rates given for each patient were recorded. The protocol stipulated a maximum infusion rate of 0.8 mg/kg/h for cipepofol, if the target RASS score was not reached, the patient would be excluded and sedatives would be given at the discretion of treating physicians.

Remifentanil was infused for analgesia before cipepofol administration, which dose was started at 0.01 μg/kg/min and adjusted to achieve a Critical-care Pain Observation Tool (CPOT) score of 0 to 1 (24). Because enrolled patients in our study suffered from major painful stimuli, including surgical wounds, and stimuli from endotracheal tubes and artificial ventilation. Experimental and clinical studies have suggested that pain influences respiration in some ways (25). To balance the bias caused by pain at baseline, we performed the goal-directed minimization of the analgesics to rule out pain-induced changes in breathing patterns, respiratory drive, and inspiratory effort. In our clinical treatment, we adhered to the eCASH concept that emphasizes early comfort using analgesia, and remifentanil is arguably one of the most commonly used opioids for acute pain in our unit, so we have chosen remifentanil to balance the pain levels at baseline.

### Data collection

Baseline data collection includes demographic data (age, sex, BMI), history of hypertension, information about the surgery (duration of surgery, American Society of Anesthesiologists physical status classification system (ASA) score, emergency surgery or elective surgery), illness severity (baseline Acute Physiology and Chronic Health Evaluation-II (APACHE-II) and Sequential Organ Failure Assessment (SOFA) score on the day of enrollment), Glasgow coma scale (GCS)-(Eye, Verbal, Motor) score after waking up from general anesthesia, time from ICU admission to inclusion, site of tracheal (oral or nasal), ventilator parameter settings [pressure support (PS) levels, positive end-expiratory pressure (PEEP), fraction of inspired oxygenation (FiO_2_)], the maintenance infusion rate of remifentanil, baseline arterial blood gas analysis.

During the intervention period, all patients were connected to a ventilator (Dräger Evita Infinity V500, Drägerwerk Verwaltungs AG, Germany) in pressure support mode. PS level was adjusted to obtain a tidal volume (VT) between 6 and 8 mL/kg of ideal body weight and a respiratory rate (RR) lower than 30 breaths/min; PEEP and FiO_2_ were adjusted to obtain arterial partial pressure of oxygen (PaO_2_) values higher than 90 mmHg. Respiratory parameters include breathing patterns [RR, VT, and minute ventilation (V_min_)], respiratory drive [airway occlusion pressure at 100 msec (P_0.1_)] (26), and inspiratory effort [pressure muscle index (PMI) (27) and expiratory occlusion pressure (P_occ_) (28)] were obtained from the ventilator. Ten consecutive respiratory cycles were averaged to determine RR, TV, and V_min_. P_0.1_, PMI, and P_occ_ were evaluated in triplicate at 20-s intervals and the average values were reported, respectively.

Cardiorespiratory parameters including [SBP and diastolic blood pressure (DBP), MAP, HR, SpO_2_, and end-tidal carbon dioxide (ETCO_2_)] were monitored continuously with Mindray monitor (BeneVision N17). ETCO_2_ will be monitored continuously using a sidestream device, and the airway adapter will be placed at the end of the endotracheal tube (DRYLINE^™^ II Water Trap, Adult).

Before starting the infusion and 30 ± 5 min after each increase in the infusion rate of cipepofol, respiratory variables, cardiorespiratory variables, and RASS scores were recorded and stored on a dedicated personal computer for further analysis. Adverse events were recorded which included bradycardia (HR < 50 beats/min); hypotension (SBP < 90 mmHg after appropriate intravenous volume replacement); apnea (respiratory rate < 8 breaths/min) or hypoxemia (SpO_2_ < 90%).

### Outcomes

The primary outcome was the change from baseline in respiratory variables (RR, VT, V_min_, P_0.1_, PMI, P_occ_) at 30 min after continuous infusion of cipepofol at 0.3 mg/kg/h.

The secondary outcomes were: (1) changes in respiratory variables (RR, VT, V_min_, P_0.1_, PMI, P_occ_) at infusion rates of cipepofol from 0.3 to 0.8 mg/kg/h; (2) changes in cardiorespiratory variables (SBP, DBP, MAP, HR, SpO_2_, EtCO_2_) at infusion rates of cipepofol from 0.3 to 0.8 mg/kg/h; (3) changes in RASS scores at infusion rates of cipepofol from 0.3 to 0.8 mg/kg/h.

### Statistical analysis

Categorical variables were described as numbers (percentages). Continuous variables were described as mean ± standard deviation (SD) and medians with interquartile range [IQR]. For primary outcome analysis, the normal data was analyzed using a paired t-test, and the non-normally distributed data was analyzed with Wilcoxon signed ranks test. For secondary outcome analysis, the changes in respiratory variables, cardiorespiratory variables, and RASS scores at infusion rates of cipepofol from 0.3 to 0.8 mg/kg/h were analyzed with a linear mixed effects model, where the dose was considered as the fixed effect, the subject as the random effect and baseline as the covariate.

As patients reached the rate of 0.8 mg/kg/h was small (only 5 patients), we performed a *post hoc* analysis to analyze the changes in respiratory variables, cardiorespiratory variables, and RASS scores as the infusion rate of cipepofol increasing from 0.3 to 0.7 mg/kg/h, with methods similarly to those secondary outcomes. In addition, we conducted a *post hoc* analysis to illustrate the changing trend in respiratory variables, cardiorespiratory variables, and RASS scores at infusion rates of cipepofol from 0.3 to 0.8 mg/kg/h with data from five patients who reached the rate of 0.8 mg/kg/h. A *p*-value of <0.05 was considered statistically significant. All analyses were performed using IBM SPSS Statistics V.26.0 and GraphPad Prism V.9.0 statistical software.

## Results

### Baseline characteristics of patients

The clinical characteristics of the patients are presented in Table 1. We enrolled 20 patients whose main diagnoses were intracranial tumors and the main causes for ICU admission were intracranial tumor resections. Remifentanil was used to relieve the pain and discomfort at baseline, whose median infusion rate for CPOT 0 to 1 was 0.01 μg/kg/min.

**Table 1:** Clinical characteristics of patients.

Variables	*N* = 20
Age, year	49.1 ± 11.7
Sex, female	16 (80%)
Body mass index, kg/m^2^	24.3 ± 2.8
History of hypertension	5 (25%)
APACHE-II	8.5 ± 3.2
Sequential Organ Failure Assessment	1 [0–3]
Emergency surgery	2 (10%)
ASA	2 [2–2]
Duration of surgery, hour	5.5 ± 2.1
GCS (Eye) after waking up from general anesthesia	4 [3–4]
GCS (Motor) after waking up from general anesthesia	6 [6–6]
Time from ICU admission to inclusion, hour	9.0 [7.3–10.0]
Site of tracheal intubation, oral	7 (35%)
Site of tracheal intubation, nasal	13 (65%)
Pressure support, cmH_2_O	5 [5–6]
Positive end-expiratory pressure, cmH_2_O	5 [5–5]
PaO_2_/FiO_2_	388.9 ± 115.7
Remifentanil infusion rate, μg/kg/min	0.01 [0.01–0.01]

Data are shown as mean ± standard deviation, medians [interquartile range], or numbers (%). All included patients’ GCS (verbal) terms were intubated and were marked as T. APACHE-II, Acute Physiology and Chronic Health Evaluation-II; ASA, American Society of Anesthesiologists; GCS, Glasgow Coma Scale; ICU, Intensive Care Unit; PaO_2_/FiO_2_, Arterial Partial Pressure of Oxygen divided by Fraction of Inspired Oxygen.

### Primary outcomes

**Figure 3:**
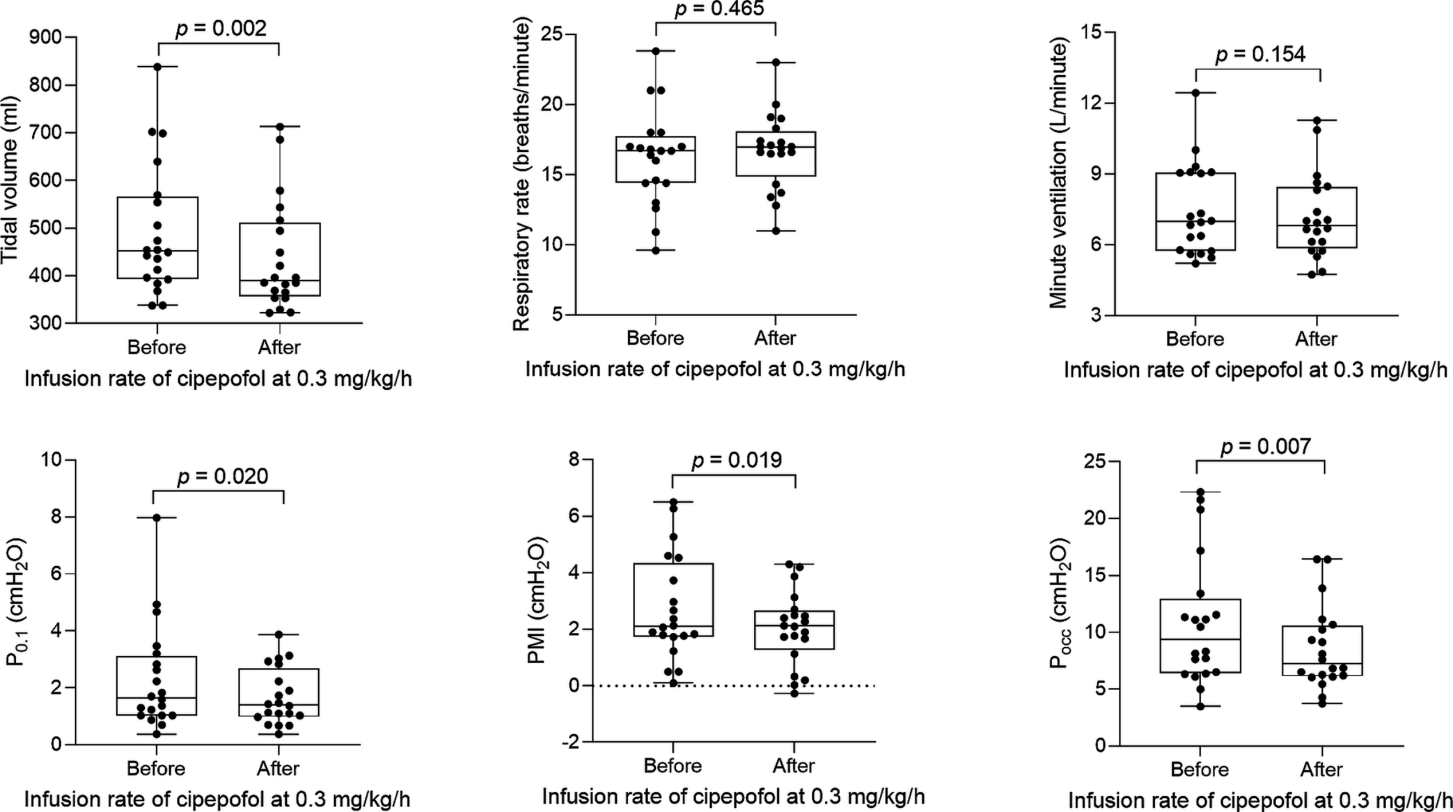
Changes in respiratory variables after 0.3 mg/kg/h cipepofol infusion for 30 min. Overlaid Box-and-whisker and scatter plot. Boxes represent the median with an interquartile range; whiskers extend the minimum and maximum values; Circles indicate individual observations; brackets denote the statistical difference between before and after infusion of cipepofol at the rate of 0.3 mg/kg/h; *p* values are shown above the brackets. P_0.1_, Airway Occlusion Pressure at 100mesc; PMI, Pressure Muscle Index; P_occ_, Expiratory Occlusion Pressure.

The titration method of cipepofol is described in Figure 2A. An initial infusion rate of cipepofol at 0.3 mg/kg/h was completed in all patients, and all patients achieved the RASS score −2 to +1. Compared with baseline values where no sedation was used, the common features of changes in main respiratory variables evaluated 30 min after infusion of cipepofol at 0.3 mg/kg/h were: a profound initial reduction in tidal volume (median 390.9, IQR [356.6–511.0] vs. 451.6 [393.5–565.9], *p* = 0.002), a non-significant change in respiratory rate (mean 16.7 ± SD 2.7 vs. 16.2 ± 3.4, *p* = 0.465), and that the change in minute ventilation was not significant (7.2 ± 1.8 vs. 7.5 ± 1.9, *p* = 0.154). For respiratory drive and inspiratory effort, there were significant reductions in P_0.1_ (1.4 [1.0–2.7] vs. 1.7 [1.0–3.1], *p* = 0.020), PMI (2.1 [1.3–2.7] vs. 2.1 [1.7–4.3], *p* = 0.019) and P_occ_ (7.2 [6.1–10.6] vs. 9.4 [6.4–12.9], *p* = 0.007) (Table 2; Figure 3).

**Table 2:** Changes in respiratory variables after 0.3 mg/kg/h cipepofol infusion for 30 min.

Variables	At baseline	After infusion	*p-*value
Tidal volume, ml	451.6 [393.5–565.9]	390.9 [356.6–511.0]	0.002
Respiratory rate, breaths/min	16.2 ± 3.4	16.7 ± 2.7	0.465
Minute ventilation, L/min	7.5 ± 1.9	7.2 ± 1.8	0.154
P_0.1_, cmH_2_O	1.7 [1.0–3.1]	1.4 [1.0–2.7]	0.020
PMI, cmH_2_O	2.1 [1.7–4.3]	2.1 [1.3–2.7]	0.019
P_occ_, cmH_2_O	9.4 [6.4–12.9]	7.2 [6.1–10.6]	0.007

Data are shown as mean ± standard deviation or medians [interquartile range]. P_0.1_, Airway Occlusion Pressure at 100mesc; PMI, Pressure Muscle Index; P_occ_, Expiratory Occlusion Pressure.

### Secondary outcomes

Due to the obvious heterogeneity in individual sensitivity to cipepofol, there was a significant difference in the final infusion rates reached. The number of patients decreased with increasing cipepofol infusion rates, with oversedation (RASS ≤ −4) being the primary cause for discontinuation. Reasons for discontinuation and final given rates are described in Figure 2B.

**Figure 4:**
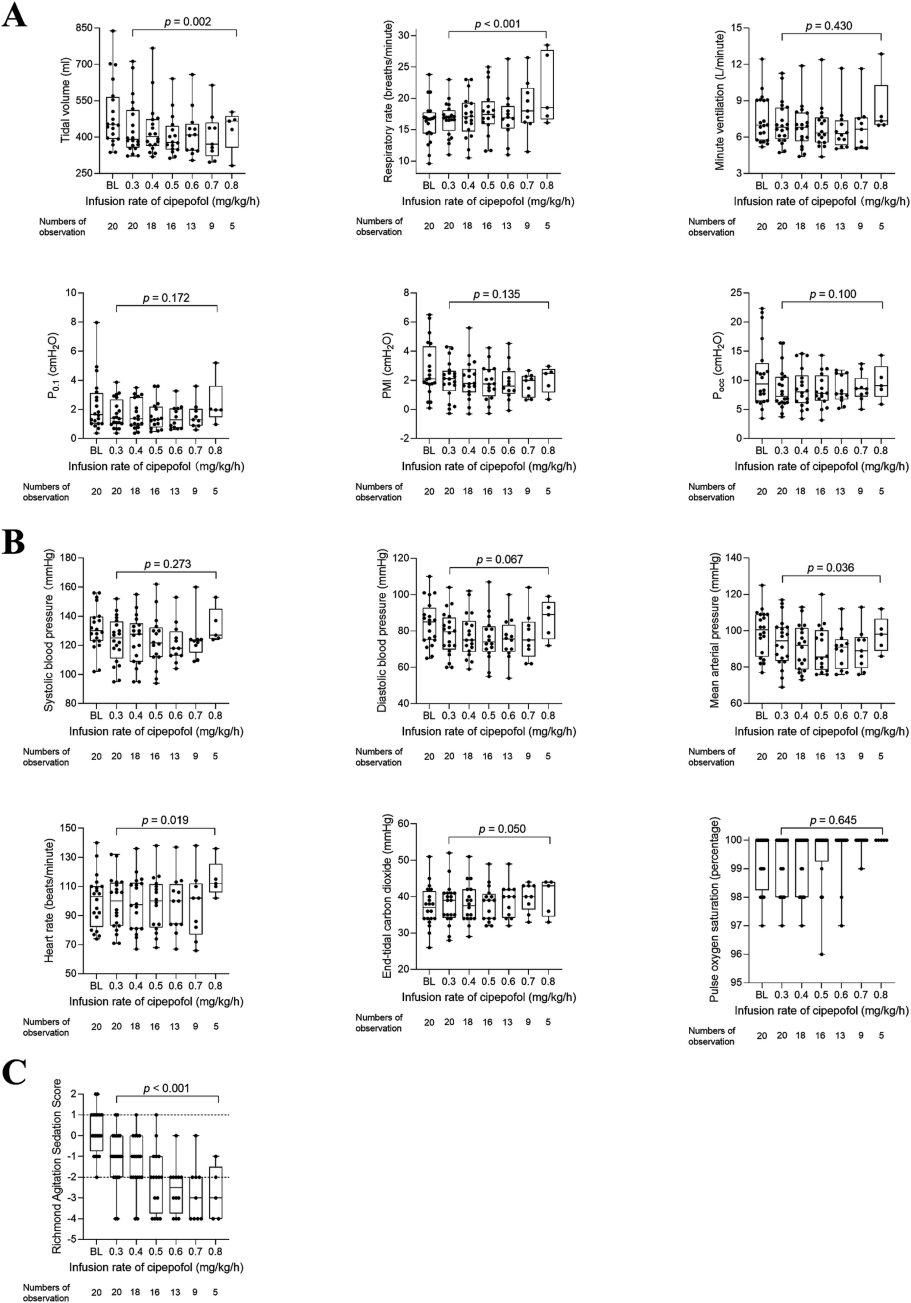
Changes in respiratory variables **(A)**, cardiorespiratory variables **(B)**, and RASS scores **(C)** from 0.3 to 0.8 mg/kg/h of cipepofol. Overlaid Box-and-whisker and scatter plot. Boxes represent the median with an interquartile range; whiskers extend the minimum and maximum values; circles indicate individual observations; brackets denote the statistical difference at different infusion rates of cipepofol (0.3–0.8 mg/kg/h); *p* values are shown above the brackets. BL, baseline; P_0.1_, Airway Occlusion Pressure at 100mesc; PMI, Pressure Muscle Index; P_occ_, Expiratory Occlusion Pressure.

The changes in respiratory variables, cardiorespiratory variables, and RASS scores at infusion rates of cipepofol from 0.3 to 0.8 mg/kg/h are shown in Table 3 and Figure 4. VT decreased further from the lower to the higher infusion rate (*p* = 0.002), with RR increasing significantly as the infusion rate increased (*p* = 0.001), and V_min_ did not change significantly (*p* = 0.430). Respiratory drive and inspiratory effort, measured by P_0.1_ (*p* = 0.172), PMI (*p* = 0.135), and P_occ_ (*p* = 0.100) showed no significant changes with increasing infusion rate. Regarding cardiorespiratory variables, there were no significant changes in SBP (*p* = 0.273) and DBP (*p* = 0.067) at higher infusion rates. However, there was a significant but small reduction in MAP (*p* = 0.036) and an increase in HR (*p* = 0.019) at higher infusion rates of cipepofol. ETCO_2_ (*p* = 0.050) showed slight changes, and SpO_2_ (*p* = 0.645) fluctuated around baseline values, consistently remaining above 96%. As the cipepofol dose increased, there was a corresponding increase in sedation depth, reflected by a decrease in RASS scores (*p* < 0.001), despite notable heterogeneity among individuals.

**Table 3:** Changes in respiratory variables, cardiorespiratory variables, and RASS scores at infusion rates of cipepofol from 0.3 to 0.8 mg/kg/h.

	Variables	Baseline values	Infusion of cipepofol at different rates (mg/kg/h)						*p* value
			0.3	0.4	0.5	0.6	0.7	0.8	
	Numbers	20	20	18	16	13	9	5	-
Respiratory variables	VT	451.6 [393.5–565.9]	390.9 [356.6–511.0]	402.6 [364.5–473.8]	377.9 [349.1–446.4]	408.8 [344.0–454.5]	370.7 [322.0–460.0]	466.1 [356.8–487.4]	0.002
	RR	16.2 ± 3.4	16.7 ± 2.7	17.2 ± 3.5	17.8 ± 3.9	17.1 ± 3.9	18.7 ± 4.3	21.4 ± 5.8	<0.001
	V_min_	7.0 [5.7–9.1]	6.8 [5.8–8.4]	6.8 [5.7–8.0]	6.3 [5.6–7.6]	6.3 [5.4–7.4]	6.6 [5.1–7.6]	7.3 [7.0–10.3]	0.430
	P_0.1_	1.7 [1.0–3.1]	1.4 [1.0–2.7]	1.4 [0.9–2.8]	1.3 [0.8–2.2]	1.3 [0.9–2.0]	1.3 [0.9–2.0]	2.0 [1.5–3.6]	0.172
	PMI	2.7 ± 1.9	2.0 ± 1.3	2.0 ± 1.4	1.8 ± 1.2	1.9 ± 1.3	1.7 ± 0.8	2.1 ± 0.9	0.135
	P_occ_	9.4 [6.4–12.9]	7.2 [6.1–10.6]	8.0 [6.1–10.8]	7.8 [6.5–10.8]	7.7 [6.7–11.2]	8.5 [7.2–10.4]	9.1 [7.2–12.4]	0.100
Cardiorespiratory variables	SBP	132 ± 15	124 ± 16	124 ± 17	123 ± 18	121 ± 14	124 ± 15	133 ± 12	0.273
	DBP	84 ± 12	78 ± 12	78 ± 12	76 ± 13	75 ± 12	77 ± 13	86 ± 11	0.067
	MAP	99 ± 13	94 ± 13	91 ± 12	90 ± 13	90 ± 11	90 ± 12	98 ± 10	0.036
	HR	100 ± 18	98 ± 18	99 ± 19	98 ± 19	98 ± 19	97 ± 23	115 ± 13	0.019
	SpO_2_	100 [98–100]	100 [98–100]	100 [98–100]	100 [99–100]	100 [100–100]	100 [100–100]	100 [100–100]	0.645
	EtCO_2_	37.2 ± 5.7	38.0 ± 5.7	38.0 ± 5.5	38.1 ± 4.8	39.0 ± 4.8	39.9 ± 3.9	40.0 ± 5.1	0.050
Sedation level	RASS	0[−1–1]	−1[−2–0]	-1[−2–0]	−2[−4--1]	−3[−4--2]	−3[−4--2]	−3[−4--2]	<0.001

Data are shown as mean ± standard deviation or medians [interquartile range]. VT, Tidal Volume; RR, Respiratory Rate; V_min_, Minute Ventilation; P_0.1_, Airway Occlusion Pressure at 100mesc; PMI, Pressure Muscle Index; P_occ_, Expiratory Occlusion Pressure; SBP, Systolic Blood Pressure; DBP, Diastolic Blood Pressure; MAP, Mean Arterial Pressure; HR, Heart Rate; SpO_2_, Pulse Oxygen Saturation; EtCO_2_, End-tidal Carbon Dioxide; RASS, Richmond Agitation Sedation Scale.

### *Post hoc* analysis

**Figure 5:**
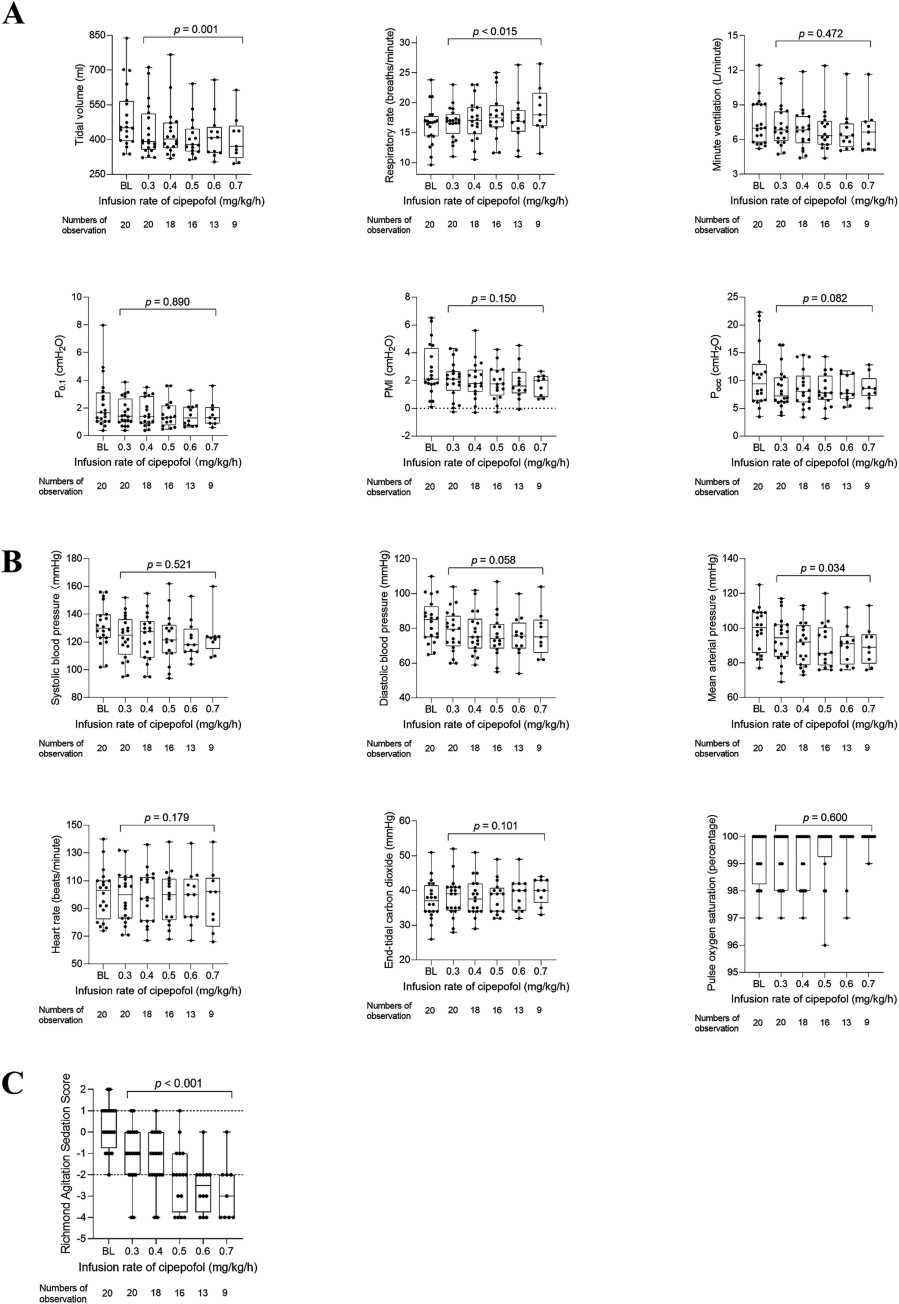
*Post hoc* analysis for changes in respiratory variables **(A)**, cardiorespiratory variables **(B)**, and RASS scores **(C)** from 0.3 to 0.7 mg/kg/h of cipepofol. Overlaid box-and-whisker and scatter plot. Boxes represent the median with an interquartile range; whiskers extend the minimum and maximum values; circles indicate individual observations; Brackets denote the statistical difference at different infusion rates of cipepofol (0.3–0.7 mg/kg/h); *p* values are shown above the brackets. BL, baseline; P_0.1_, Airway Occlusion Pressure at 100mesc; PMI, Pressure Muscle Index; P_occ_, Expiratory Occlusion Pressure.

Due to only five patients reaching the final infusion rate of 0.8 mg/kg/h, we conducted a *post hoc* analysis to analyze changes in respiratory variables, cardiorespiratory variables, and RASS scores as the cipepofol infusion rate increased from 0.3 to 0.7 mg/kg/h (Figure 5). The observed trends in all measured variables were similar to those when data at 0.8 mg/kg/h were included except for HR. In *post hoc* analysis, there was no significant difference in HR with increasing infusion rate of cipepofol from 0.3–0.7 mg/kg/h (*p* = 0.179).

**Figure 6:**
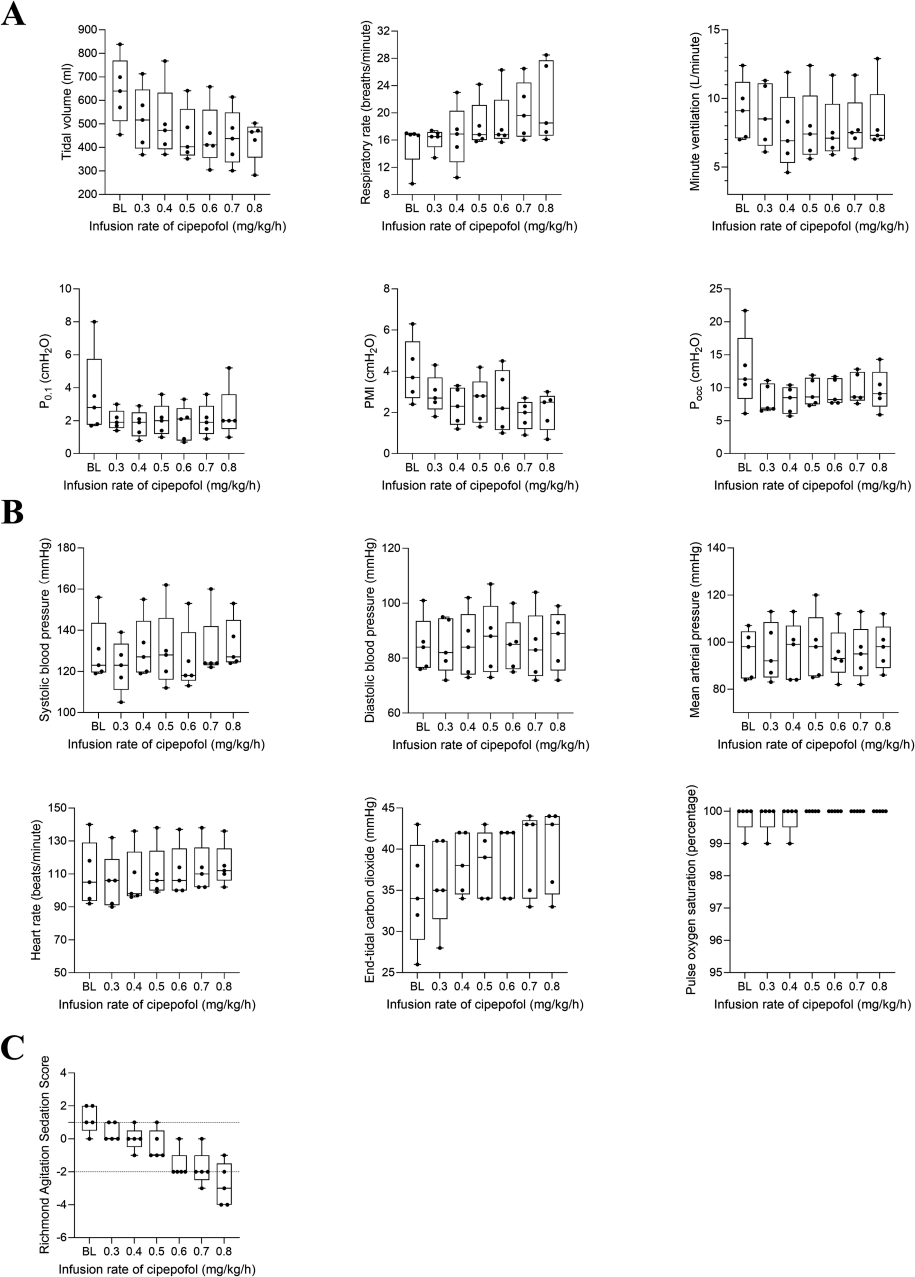
*Post hoc* analysis for changing trend in respiratory variables **(A)**, cardiorespiratory variables **(B)**, and RASS scores **(C)** from 0.3 to 0.8 mg/kg/h of cipepofol. Data were obtained from five patients who reached the maximal infusion rate of 0.8 mg/kg/h. Overlaid box-and-whisker and scatter plot. Boxes represent the median with an interquartile range; whiskers extend the minimum and maximum values; circles indicate individual observations; BL, baseline; P_0.1_, Airway Occlusion Pressure at 100mesc; PMI, Pressure Muscle Index; P_occ_, Expiratory Occlusion Pressure.

Then, the changing trend of respiratory variables, cardiorespiratory variables, and RASS scores for 5 patients who reached the maximum infusion rate of 0.8 mg/kg/h were displayed in Figure 6. The primary characteristics included a decrease in VT, an increase in RR, and no significant change in V_min_, P_0.1_, PMI, and P_occ_ as the infusion rate increased. The RASS scores decreased significantly and cardiorespiratory variables did not change significantly.

## Discussion

We conducted a physiological study to investigate the effects of cipepofol on breathing patterns, respiratory drive, and inspiratory effort measurements. We chose 0.3 mg/kg/h as the initial infusion rate for sedation, which was based on the multicenter studies in which we have participated (17), the study concluded that for patients receiving mechanical ventilation, the median maintenance dose of cipepofol to achieve the RASS score − 2 to +1 was 0.3 mg/kg/h. According to the above study’s conclusion from multiple ICU units including our clinical experience, we have chosen 0.3 mg/kg/h as the initial infusion rate and the primary respiratory effects observation time.

In mechanically ventilated patients, a notable decrease in tidal volume was observed following the administration of cipepofol, with a more pronounced effect noted at a higher infusion rate, which was similar to that when propofol was used for the induction and maintenance of general anesthesia (10). However, the exact mechanism underlying the decreased tidal volume induced by cipepofol remains unclear. Studies have demonstrated propofol reduces tidal volume by depressing phrenic nerve activities and diaphragmatic movement (29, 30). Further mechanistic studies are necessary to elucidate the effect of cipepofol.

Upon initiating the continuous infusion of cipepofol, we observed an immediate decrease in tidal volume with no significant change in respiratory rate. As the cipepofol infusion rate increased, tidal volume continued to decrease, followed by an obvious increase in respiratory rate. This pattern of changes mirrors that observed with propofol, which has been shown to increase respiratory rate as a compensatory mechanism (31). Ventilation is typically measured through tidal volume and respiratory rate, with minute ventilation derived from these parameters (32). Over the intervention period, the net effect of changes in tidal volume and respiratory rate was that minute ventilation remained unchanged significantly.

There was a significant reduction in respiratory drive represented by P_0.1_ and inspiratory effort represented by PMI and P_occ_ after the infusion of cipepofol at 0.3 mg/kg/h. Studies have shown that “behavioral” factors (anxiety, agitation) modulate the activity of the respiratory centers (33). In our study, the initial dose achieved satisfactory sedation (RASS -2 to +1) and relieved the increased respiratory drive and inspiratory effort caused by these behavioral factors. Notably, a higher dose of cipepofol did not lead to further significant change in these indices, consistent with findings from a previous study that reported no correlation between deeper sedation and lower P_0.1_ (34). On one side, decreased respiratory effort from sedation can contribute to disuse atrophy and dysfunction of the diaphragm (35). On the other side, sedation could reduce respiratory effort and high tidal volume, mitigating the risk of patient self-inflicted lung injury (P-SILI) and diaphragm injury from excessive ventilatory effort (36). Therefore, clarifying the effects of cipepofol on respiratory drive and inspiratory effort, including P_0.1_, PMI, P_occ_ should be a priority in bedside care.

At an infusion rate of 0.3 mg/kg/h, cipepofol achieved a sedation level of RASS score − 2 to +1, with two patients experiencing oversedation (RASS ≤ −4) in our study. Increasing the infusion rate led to deeper sedation, though individual sensitivity to cipepofol varied, resulting in a wide range of final infusion rates. Hemodynamic stability was maintained with minimal fluctuations in SBP, DBP, and MAP, consistent with previous studies (37). No drug-related bradycardia was observed, instead, a faster heart rate was observed at higher infusion rates, especially at the rate of 0.8 mg/kg/h. However, a *post hoc* analysis showed a non-significant change in heart rate with an increasing infusion rate of cipepofol from 0.3 to 0.7 mg/kg/h. The reason might be explained as the heart rate was inherently faster in five patients who reached the 0.8 mg/kg/h infusion rate. Sinus tachycardia has also been reported with cipepofol used for anesthesia induction in elective surgery (38). Further investigation is needed to understand the effects of cipepofol on heart rate and its exact mechanism in patients under neurological surgery. There were no adverse effects including propofol infusion syndrome (PRIS) during the intervention period.

### Limitations of the study

First, we performed goal-directed minimization of the analgesics with remifentanil, which may confound the effect of cipepofol on respiration. Under similar circumstances, to explore the effects of propofol on respiration, Liu L et al. maintained a continuous infusion of analgesics during the study period (39). Moreover, we titrated the infusion rate of remifentanil for CPOT 0–1. After achieving the goal, the remifentanil infusion rate would not be changed throughout the study period. Nevertheless, we were unable to remove the possible effect of baseline analgesia. Second, the study was single-center with a small sample size, and all enrolled patients were neurological surgery patients, which restricted the generalization of our conclusions to all patients. Lastly, the accuracy of non-invasive respiratory drive and inspiratory drive indices, including P_0.1_, PMI, and P_occ_ may be questioned. Although invasive measures using esophageal pressure are more accurate, they are technically challenging and not widely available. Our findings in this area should be considered hypothesis-generating and warrant further validation.

## Conclusion

The main finding was that cipepofol demonstrates the capability to reach a satisfactory sedation level in mechanically ventilated adult patients. The primary effect of cipepofol on breathing patterns was a decrease in tidal volume, while changes in respiratory rate and minute ventilation were insignificant. The effect on respiratory drive and inspiratory effort significantly reduced P_0.1_, PMI, and P_occ_.

## Data Availability

The raw data supporting the conclusions of this article will be made available by the authors, without undue reservation.
